# Mechanism of Tungsten Film Adhesion Enhancement on Alumina Ceramics via Microgroove Spacing During Multi-Abrasive Scratching

**DOI:** 10.3390/mi17040465

**Published:** 2026-04-11

**Authors:** Xue Yang, Jiayi Wu, Wenlong Liu, Wenhao Ma, Chen Jiang

**Affiliations:** School of Mechanical Engineering, University of Shanghai for Science and Technology, Shanghai 200093, China; wu_jyi@163.com (J.W.); 243391635@st.usst.edu.cn (W.L.); 255381547@st.usst.edu.cn (W.M.); jc_bati@163.com (C.J.)

**Keywords:** alumina ceramic, microgroove spacing, interfacial adhesion, multi-abrasive scratching, stress distribution

## Abstract

During the high-temperature deposition of tungsten thin films on alumina ceramic substrates, the inherent mismatch in thermal expansion coefficients frequently triggers interfacial delamination, where uncontrollable factors in stochastic surface topographies can exacerbate localized stress concentrations. To resolve these interfacial failures, the enhancement of interfacial adhesion through a deterministic surface microgroove design is identified as the general objective of the present research. Within this framework, the establishment of a robust quantitative mapping between the transverse scratching offset distances and the resultant periodic microgeometry is first pursued as a specific experimental objective. This methodological approach effectively transforms the stochastic nature of the substrate into deterministic geometric configurations. Second, a specific numerical objective is fulfilled by evaluating the interfacial stress redistribution and damage evolution utilizing refined thermomechanical coupled simulations based on the cohesive zone model. The integrated findings demonstrate that optimizing the microgroove spacing effectively governs the morphological transition and broadens stress diffusion pathways to mitigate thermal mismatch effects. Specifically, the structural optimization at a spacing of 28.8 µm facilitates an approximately 31.8% reduction in the maximum interfacial stress and a 10% decrease in the average film stress compared to the 13.6 µm spacing. Finally, this research clarifies the underlying mechanisms of stress buffering and provides a rigorous engineering methodology for the structural design of reliable high-performance ceramic–metal interfaces in extreme environments.

## 1. Introduction

Alumina ceramics, as a representative hard and brittle material, exhibit high hardness, high strength, a low coefficient of thermal expansion (CTE), and excellent chemical stability [1,2]. These properties allow them to maintain structural stability under harsh high-temperature conditions [3]. In semiconductor manufacturing, high-temperature thin-film deposition is a key process that relies on ceramic components with high thermal stability, which are integral to deposition equipment [4]. Beyond general deposition equipment, these high-performance ceramic metal components are critically applied in specialized fields of applications such as electrostatic chucks, wafer heating stages, and aerospace thermal barrier systems, where extreme thermal stability and robust interfacial adhesion are required [5,6].

Enhancing the adhesion reliability of ceramic–metal thin-film interfaces can be achieved by introducing surface microstructures on the substrate to strengthen mechanical interlocking between the film and the substrate [7,8]. Such designs effectively mitigate stress concentration and the accumulation of residual thermal stress arising from the mismatch in the coefficient of thermal expansion (CTE). Under high thermal conductivity conditions, residual stresses induced by thermal expansion reduce the crack initiation threshold, increasing the susceptibility of thin films to cracking [9]. Finite element analysis (FEA) combined with the cohesive zone model (CZM) has demonstrated that interfacial stress concentration is a dominant factor governing thin-film failure. Modifying coating thickness and cohesive strength can effectively relieve this stress and improve film durability [10]. Simulation studies have further examined the evolution of thermal stress in tungsten coatings under high heat flux conditions and have shown that grain boundary reconstruction and crack propagation can accelerate film failure. In addition, surface roughening has been shown to mitigate tensile stresses induced by the mismatch in the coefficient of thermal expansion (CTE) [11,12].

Surface microstructure modification has attracted increasing attention as an effective approach for optimizing interfacial performance. A moderate level of surface roughness can significantly enhance mechanical interlocking between the thin film and the substrate, leading to an increased load-bearing capacity of the interface. However, excessive roughness may magnify stress concentration, making it necessary to balance surface roughness and stress distribution in surface microstructure design [8]. Previous studies have investigated the optimization of thin-film adhesion through various surface treatment techniques and microstructural designs. Within the state of the art, these techniques encompass both conventional and non-conventional alternative methods. Conventional approaches such as mechanical sandblasting and chemical etching are widely utilized in industry but they often struggle to maintain precise geometric control over the microstructures [13]. Consequently, non-conventional surface modification methods have emerged as cutting-edge solutions. Wang et al. [14] utilized the Rotational Ultrasonic Vibration-assisted Machining Technique (RUVT) to generate microtextures, thereby improving coating adhesion by enhancing both wettability and mechanical interlocking. Similarly, Zheng et al. [15] doubled the critical load of coatings by laser texturing the alumina surface, which was attributed to enhanced mechanical interlocking. Although these methods have demonstrated improvements in film adhesion, they generally involve complex and high-cost processes, and their performance under high-temperature or high-load conditions remains insufficiently validated. In numerical simulation studies, experimental investigations have been combined with modeling to analyze stress evolution at the film–substrate interface under thermomechanical coupling. Long et al. [16] investigated the influence of ceramic coating thickness on the delamination mechanism, revealing how an increase in coating thickness can shift the failure mode from coating cracking to interfacial peeling. Furthermore, Liu et al. [17,18] explored interface delamination and buckling of thin films under wedge indentation using the Cohesive Zone Model (CZM) and Finite Element Analysis (FEA), further elucidating the roles of interfacial strength and energy in the initiation of peeling. These studies offer theoretical guidance for optimizing roughness and periodic texture regulation under thermomechanical coupling by linking microscopic textures with macroscopic adhesion performance [19,20].

Despite these advancements, existing numerical simulations rarely address multi-scratch interaction mechanisms or the influence of scratch spacing on thin-film stress distribution, which leaves deposition stress coupled behavior insufficiently understood. Moreover, conventional roughness parameters such as *Ra* and *Rq* are insufficient to fully characterize surface geometry because they cannot establish a reliable geometric correlation for numerical stress optimization. The primary motivation for this research stems from the critical requirement to bridge this gap by replacing uncontrollable and stochastic surface topographies with a deterministic periodic microgroove design. By establishing a robust quantitative mapping between manufacturing parameters and periodic microgeometry, this study is motivated to transform unpredictable surface features into computationally reproducible structures. This approach provides a predictable and cost effective methodology for improving the damage tolerance of advanced thin-film systems under extreme thermal environments.

**Figure 1:**
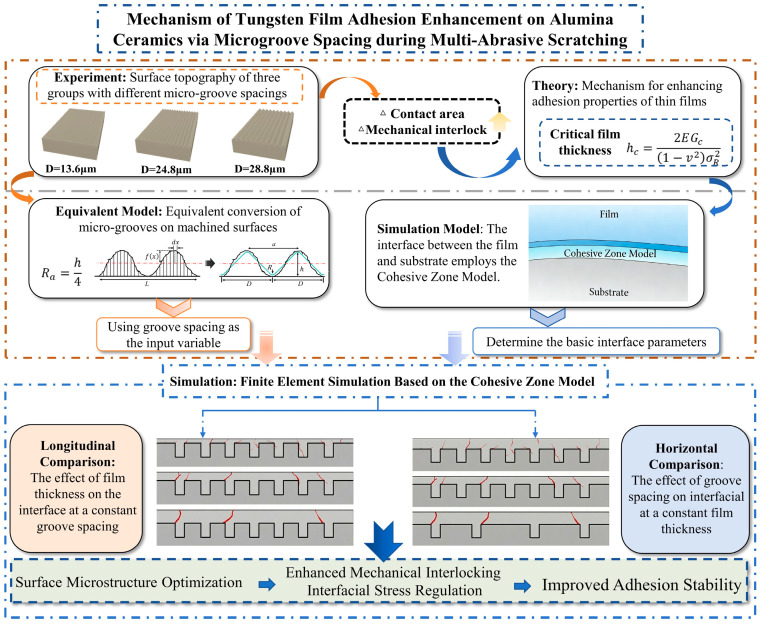
Experimental and simulation research framework.

To implement this deterministic design and investigate the resulting interfacial mechanics, the specific objectives of this study are fulfilled through three integrated tasks. Initially, diamond multi-abrasive scratching tools are designed and applied using a high precision three-axis machining center to fabricate periodic microgrooves on alumina substrates. Furthermore, a simplified finite element model is developed based on the obtained surface geometry, where the cohesive zone model (CZM) is employed to perform thermomechanical coupling simulations. Finally, the stress mitigation mechanisms and the synergistic effect of groove spacing and film thickness are quantified to provide methodological guidance for microstructure-assisted interfacial regulation. The research procedure is illustrated in Figure 1.

## 2. Experimental Procedure

### 2.1. Tools and Materials

**Figure 2:**
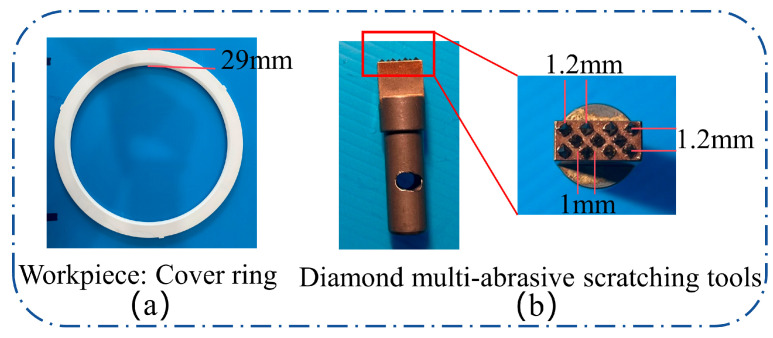
Materials and scratching tool: (**a**) Geometric morphology of the alumina ceramic substrate; (**b**) design of the multi-abrasive diamond tool.

The experiments employed an alumina ceramic cover ring as the substrate material, featuring an outer diameter of 355 mm, an inner diameter of 297 mm, and a thickness of 4 mm. The geometry is illustrated in Figure 2a. To simulate the cooperative scratching process of multiple grains, a diamond multi-abrasive scratching tool was independently designed and fabricated, as shown in Figure 2b. The selected diamond grains were pyramidal with a 1 mm diameter, 90° apex angle, and a 1.2 mm inter-grain spacing. The grains were secured to a metal base using brazing, and a high-precision surface grinding process was used to calibrate the grain heights, controlling the height tolerance to within ±2 µm to ensure the stability and repeatability of the scratching process.

### 2.2. Experimental Setup

**Figure 3:**
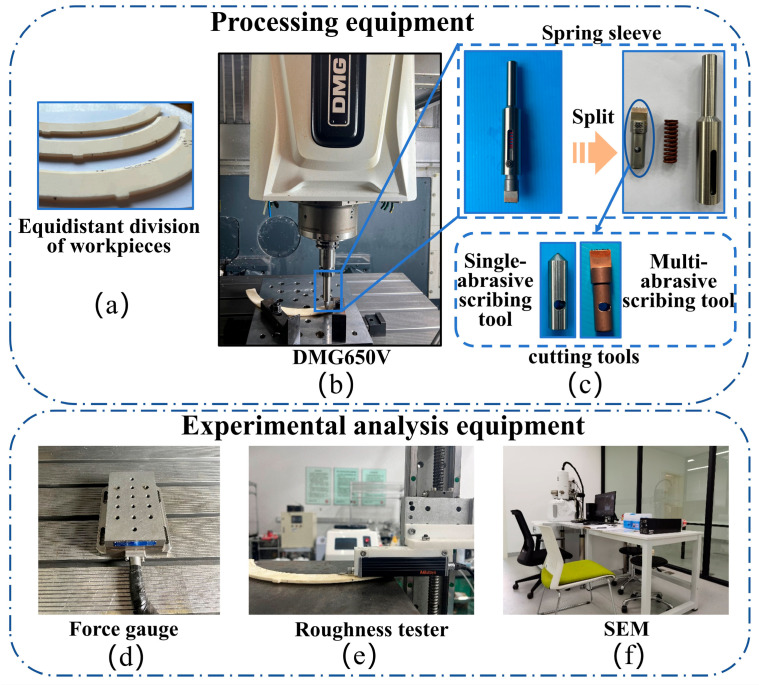
Experimental fabrication and measurement equipment: (**a**) Alumina ceramic workpiece; (**b**) DMG 650 Vertical Machining Center; (**c**) specialized spring sleeve; (**d**) Kistler 9257B three-component dynamometer; (**e**) Mitutoyo surface profiler; (**f**) Scanning Electron Microscope (SEM).

To facilitate repeatable experiments, simplify measurement, and prevent damage to the machined surfaces from repeated clamping, the alumina ceramic cover ring was segmented into three 120° equal-angle sections. The scratching experiments were conducted on a DMG 650V 3-axis Vertical Machining Center (DMG MORI, Bielefeld, Germany), as depicted in Figure 3a. This equipment utilizes a highly rigid structure that effectively minimizes mechanical vibrations during the scratching process. Equipped with a laser probe and an in-machine tool presetter, it guarantees sufficient dynamic stability and machining repeatability to fulfill the strict requirements of microgroove fabrication. A specialized sleeve fixture was designed to secure the scratching tool, as shown in Figure 3c, in order to protect the spindle system and ensure stable clamping. During the experiments, a Kistler 9257B three-component dynamometer (Kistler Group, Winterthur, Switzerland) was mounted beneath the workpiece to record real-time scratching forces, as shown in Figure 3d. After machining, a Mitutoyo surface profiler (Mitutoyo Corporation, Kawasaki, Japan) was used to measure the surface roughness and profile morphology (Figure 3e). Subsequently, the workpiece surfaces were ultrasonically cleaned to remove debris, and a Scanning Electron Microscope (TESCAN ORSAY HOLDING, Brno, Czech Republic) was employed to observe the surface morphology for quality assessment, as shown in Figure 3f.

### 2.3. Experimental Design Plan

To investigate the influence of process parameters on surface morphology and to identify a critical load that avoids substrate damage, single-grain scratching experiments were first performed.

In these experiments, the feed rate was fixed at 200 mm/min and the scratch length was set to 10 mm. The axial load was systematically varied (20, 40, 60, 80, 100 N) to examine its effect on groove formation, with detailed processing parameters listed in Table 1. Based on these experimental results, an appropriate critical load was selected for the subsequent multi-abrasive scratching experiments.

Subsequently, preliminary multi-abrasive scratching tests were conducted at the identified critical load of 60 N to determine the optimal parameters for investigating the regulation of interfacial adhesion via surface morphology. Based on the results, the transverse offset distances (*D*) between adjacent scratching paths were specifically set at 13.6 µm, 24.8 µm, and 28.8 µm. The selection of these precise values was fundamentally driven by the objective of establishing a deterministic correlation between target surface roughness (*Ra*) and periodic micro-geometry. Preliminary investigations revealed that the surface morphology undergoes three distinct evolutionary stages: severe inter-grain interference, transitional overlap, and morphological saturation. To capture these characteristic states, target roughness levels of approximately 1.7 µm, 3.1 µm, and 3.6 µm were identified as the representative nodes. To ensure the reproducibility of these states, the specific CNC machining parameters were calculated based on the 90° tool profile geometry and the deterministic relationship D=8Ra. The detailed mathematical derivation and the physical mechanisms governing this geometric evolution are systematically analyzed in Section 4.1.1. By utilizing these three calculated spacings, periodic microstructures with fundamentally different geometric characteristics ranging from high-overlap triangular ridges to isolated trapezoidal structures were successfully fabricated for subsequent film deposition and adhesion analysis.

### 2.4. Analysis and Measurement

The results of the single-grain scratching experiments served as the basis for identifying critical process parameters required to avoid substrate damage. The measured scratch depths under different axial loads are shown in Table 1. As the axial load increased from 20 N to 100 N, the scratch depth increased approximately linearly from 5 µm to 25 µm. However, the rate of increase in scratch depth decreased when the axial load exceeded 60 N.

Based on the results above, 60 N was selected as the critical load for the subsequent multi-abrasive scratching experiments. A series of periodic microgroove structures with different spacings were fabricated on the alumina ceramic surface by adjusting the transverse offset distance. Roughness measurements of the surfaces with different spacings (Table 2) indicated that increasing the groove spacing led to a significant enhancement in the arithmetic mean roughness (*Ra*), confirming that groove spacing is a key geometric parameter for surface morphology control.

Furthermore, the dynamic change in the axial load is a direct mechanical representation of the multi-abrasive scratching process. Its periodic fluctuation pattern is closely related to the groove spacing regulation and directly affects the quality of the resulting surface morphology. This experiment adopted a “static scratching combined with vertical offset” processing strategy, where the tool remained fixed. After completing each scratch, the tool was offset by the set distance in the vertical direction before proceeding with the next pass. During this process, each diamond grain undergoes a “plunge–steady-state cutting–exit” cycle, corresponding to one complete fluctuation period in the load signal.

For all three sets of experiments, the axial force Fz fluctuated around the preset value of 60 N. Further comparison of the force signal characteristics under different groove spacings revealed a systematic evolution in the force–time curves as the spacing increased.

**Figure 4:**
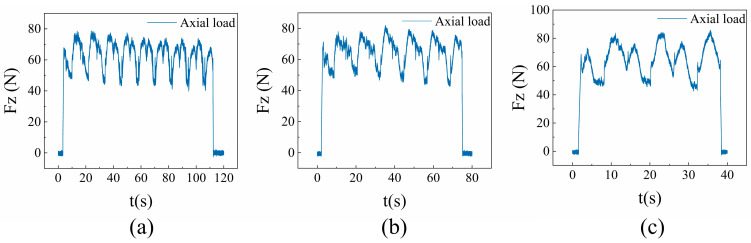
Comparison of axial load characteristics in multi-abrasive scratching under groove spacing regulation: (**a**) variation in axial load at an offset distance of 13.6 µm; (**b**) variation in axial load at an offset distance of 24.8 µm; (**c**) variation in axial load at an offset distance of 28.8 µm.

The signal in Figure 4a exhibits high-frequency and low-amplitude fluctuations, reflecting significant interference between adjacent grain paths under the smallest spacing condition, where the diamond grains undergo continuous secondary cutting of the ridge formed by the preceding groove. The load in Figure 4b fluctuates within the range of 60–80 N, with intermediate peak values and a reduced peak-to-valley difference. This indicates a reduction in inter-grain cutting interference due to the increased groove spacing, resulting in a moderate increase in the cutting load of individual grains. Conversely, the load in Figure 4c fluctuates between 50 and 90 N, featuring the highest peak values and a significant difference in amplitude. This demonstrates that inter-grain cutting interference is substantially reduced at this spacing, requiring individual grains to bear a higher cutting load. Given the hard and brittle nature of alumina ceramic, the localized stress concentration during grain plunge easily leads to an instantaneous high load, which corresponds to an increased material removal rate per cut.

## 3. Experimental Results

### 3.1. Analysis of Single-Scratch Test Results

**Figure 5:**
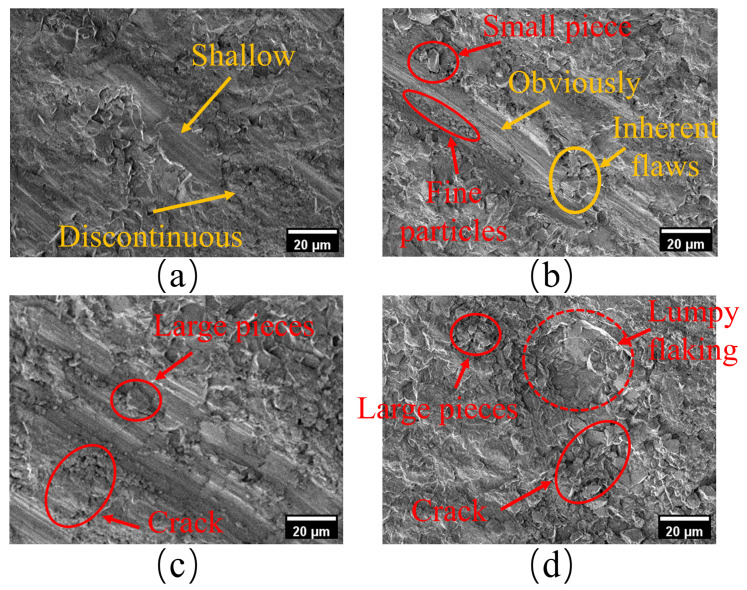
SEM micrographs of scratches on alumina ceramics under different axial loads: (**a**) Surface morphology of a single scratch under 40 N load; (**b**) surface morphology of a single scratch under 60 N load; (**c**) surface morphology of a single scratch under 80 N load; (**d**) surface morphology of a single scratch under 100 N load.

The SEM micro-morphology of the scratches on the alumina ceramic under various axial loads was observed to systematically evaluate the influence of the load on the single-scratch formation mechanism and damage behavior. The 20 N load was not illustrated as the axial force was too small to form continuous macroscopic scratches on the material surface. As shown in Figure 5, the material response progressively transitioned from localized plastic deformation to bulk brittle fracture with the increasing load.

At an axial load of 40 N, the grain cutting action was relatively weak, producing shallow and discontinuous scratches, with only faint traces of localized plastic deformation, as shown in Figure 5a. When the load was increased to 60 N, the material response was dominated by plastic flow, producing continuous, uniform scratches with distinct textures, as shown in Figure 5b. Although minor block-like spallation and fine particles are present at the groove edges, and some inherent surface defects are visible, the overall groove integrity remains good. This suggests that this load was at the critical state for plastic forming. Further increasing the load to 80 N resulted in significant stress concentration, as shown in Figure 5c. Extending cracks appear perpendicular to the scratch direction, accompanied by larger block-like spallation of the surface material, indicating an intensified degree of damage. At the high load of 100 N, the material fully entered the brittle fracture mode, resulting in large-scale spallation and cracking, accompanied by increased damage depth, as shown in Figure 5d.

### 3.2. Analysis of Multiple-Scratch Test Results

From the SEM images, it is evident that a periodic microgroove and ridge structure was formed under all three spacings. The regulation of groove spacing directly determines the degree of interference between adjacent grain scratching paths, consequently altering the cross-sectional morphology of the grooves and ridges.

**Figure 6:**
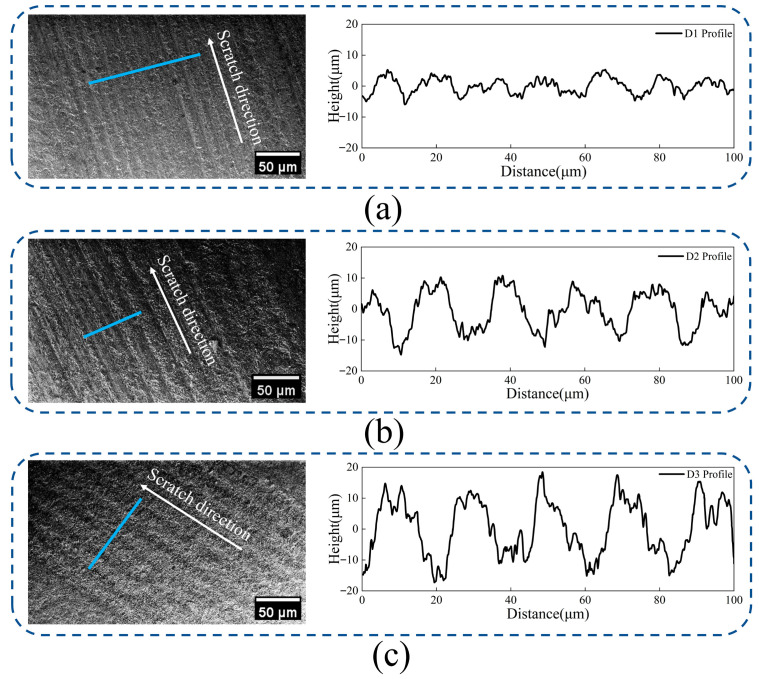
Comparison of microgroove surface topography and contour curves: (**a**) Surface topography and contour map with a groove spacing of 13.6 µm; (**b**) surface topography and contour map with a groove spacing of 24.8 µm; (**c**) surface topography and contour map with a groove spacing of 28.8 µm. The blue lines indicate the cross-sectional contour profiles.

In Figure 6a, the cross-section of the groove and ridge closely approximates an isosceles right triangle, and the high overlap between adjacent structures leads to repeated cutting of the ridge, resulting in a relatively flat and dense overall morphology with a small peak-to-valley difference in the corresponding surface profile. While this strong interference cutting at a small spacing yields better morphological uniformity, the insufficient ridge height limits the anchoring depth for mechanical interlocking. Physically, this shallow topological feature fails to provide sufficient spatial constraint to restrict the potential interfacial sliding of the deposited film under thermal mismatch.

As the spacing increases to that shown in Figure 6b, the interference effect between adjacent grain scratching areas is further reduced, and the cutting of the ridge continues to weaken. The cross-section gradually transitions from a triangular shape to an isosceles trapezoid, where both the groove depth and the ridge height reach relatively high equivalent values of approximately 12.4 µm.

With a further increase in groove spacing in Figure 6c, the groove depth and ridge height again reach high stability at 14.4 µm, but the growth rate of the roughness slows down, indicating that the morphological evolution is gradually entering a “saturation trend phase”. These observations demonstrate that precise control of substrate surface micro-morphology can be achieved through groove spacing regulation, providing an effective approach for optimizing mechanical interlocking at the film substrate interface. From a mechanistic perspective this maximized peak to valley topography functions as a series of robust physical anchors that deeply embed into the deposited film to restrict lateral displacement and fundamentally strengthen the interfacial load-bearing capacity.

## 4. Analysis and Discussion

### 4.1. Finite Element Simulation

#### 4.1.1. Creation of Microgrooves on the Substrate Surface

To analyze the effect of the alumina ceramic substrate’s surface roughness on thermal stress distribution and tungsten thin-film adhesion, a simplified model was adopted to construct the periodic microgroove structure. This approach is based on experimentally measured surface roughness parameters, ensuring a high degree of consistency between the model geometry and the actual machined morphology, while simultaneously reducing computational complexity. This simplification facilitates the investigation of the microstructure’s regulating mechanism on interfacial mechanical behavior. The surface roughness is quantified by the amplitude parameter *Ra*, defined as [21].

(1) $$Ra=\frac{1}{L}\int_{0}^{L}\left|f\left(x\right)\right|dx$$

where f(x) is the surface profile deviation function and *L* is the sampling length (or measurement length). The morphology generated by grain scratching is referred to as a “groove”, and the raised section between adjacent grooves is termed a “ridge”. To bridge the gap between macroscopic engineering measurements and microscopic geometric modeling, the profile is represented by a periodic isosceles triangle. While *Ra* serves as the primary evaluation metric in machining, the maximum profile height *Rz* is semantically equivalent to the geometric groove depth *h* in this idealized periodic model. According to the mathematical derivation for periodic triangular textures [22], a deterministic relationship exists between the measured roughness and the absolute geometric depth, which can be expressed as

(2) Rz=h=4Ra

where h is the groove depth, which is also equivalent to the ridge height. This unified correlation ensures that the numerical model maintains physical consistency with the experimental surface characterization while providing the necessary geometric constraints for subsequent stress distribution analysis.

**Figure 7:**
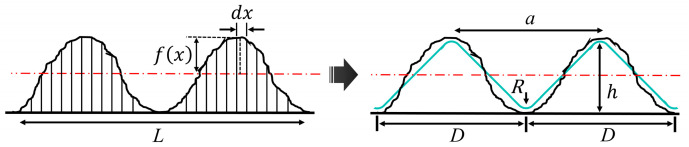
Schematic diagram of surface roughness equivalence to microgroove geometry model.

This simplification retains the periodic undulation of the surface microstructure and significantly reduces the number of finite element (FE) meshes, thereby avoiding the computational burden associated with modeling random rough surfaces. Since the grooves are generated by diamond grain scratching, and based on the pyramidal shape of the diamond grain, the groove cross-section was designed as an isosceles right triangle with an apex angle of 90°, as shown in Figure 7. Furthermore, to prevent stress singularities at sharp corners in the numerical simulation, a filet was applied to the apex of the triangle.

This fileting process does not significantly alter the overall geometric features but effectively alleviates stress concentration, ensuring that the simulation results reflect true physical phenomena rather than numerical artifacts [23]. Correspondingly, the geometric relationship dictates that the groove width equals the groove spacing *D*, and is twice the groove depth *h*. The groove depths and spacings corresponding to the three typical surface roughnesses generated in the experiment are listed in Table 3.

**Figure 8:**
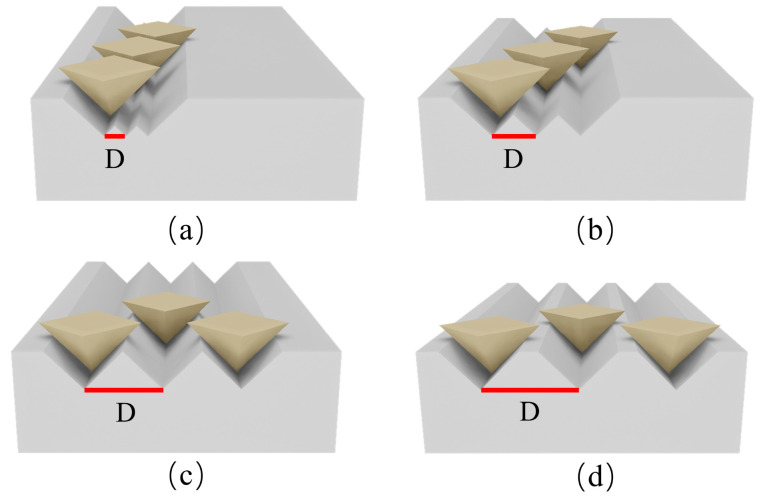
Three-dimensional schematic diagram of microgroove morphology controlled by multi-grain spacing: (**a**) Overlapping ridge subjected to secondary cutting at small groove spacing; (**b**) reduced inter-grain interference with moderate groove spacing; (**c**) groove isolation and stabilization at critical groove spacing; (**d**) ridge cross-section transition to isosceles trapezoid at maximum groove depth.

As shown in Figure 8, the geometric morphology of the grooves and ridges changes significantly with variations in the groove spacing *D*, which is the fundamental reason for the different roughness levels observed in the three machined surfaces.

During the multi-abrasive scratching process, raised ridge structures are formed between adjacent grooves. At small groove spacing, partial overlap existed between adjacent grain paths, resulting in the ridge being subjected to secondary cutting by the grains on both sides, as shown in Figure 8a. Consequently, the ridge height *h* decreases, and the ridge cross-section is approximately an isosceles right triangle, consistent with the groove. From a mechanical perspective, this severe secondary material removal flattens the interfacial topology, which restricts the maximum depth available for the thin film to physically anchor into the substrate.

At small groove spacing, partial overlap occurs between adjacent grain paths, causing the ridge to undergo secondary cutting by grains on both sides, as shown in Figure 8b. Upon reaching a critical groove spacing, further increases in *D* no longer induced interference between the grooves, leading to their progressive isolation and stabilization of the cross-sectional shape, as shown in Figure 8c.

**Figure 9:**
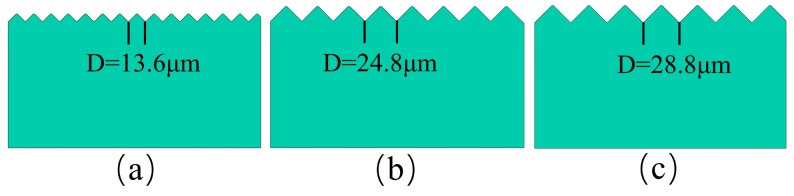
Three different finite element simulation substrates constructed based on equivalent models: (**a**) Corresponding to the substrate finite element model with a groove spacing of 13.6 µm; (**b**) corresponding to the substrate finite element model with a groove spacing of 24.8 µm; (**c**) corresponding to the substrate finite element model with a groove spacing of 28.8 µm.

At this point, the cross-section of the ridge transitioned from an isosceles triangle to an isosceles trapezoid, as shown in Figure 8d. At this stage, both groove depth and ridge height reach their maximum values and remain unchanged. Further increases in groove spacing do not lead to noticeable changes in the surface roughness *Ra*. This geometric stabilization provides a critical mechanical advantage because the fully preserved trapezoidal ridges maximize the available interlocking volume, which directly enhances the load-bearing capacity and the overall structural integrity of the film substrate interface. This analysis explains why different groove spacings result in distinct groove and ridge cross-sectional morphologies under the same axial load of 60 N. In summary, this study constructed the three different substrate models required for finite element simulation, based on the actual machined surfaces, as illustrated in Figure 9.

#### 4.1.2. Finite Element Simulation of Thermal Mismatch

To investigate the influence of thermal mismatch stress on the interfacial performance between the tungsten thin film and the alumina ceramic substrate, this section conducts a thermomechanical coupled finite element analysis based on the surface microgroove model established in Section 4.1.1. The simulation focuses on the accumulation of tensile stress in the film, localized stress concentration, and interfacial stress evolution induced during the deposition heating process due to the mismatch in the coefficients of thermal expansion (CTEs) between tungsten and alumina. The objective is to clarify the role of periodic microgroove structures in regulating thermal mismatch effects at the film–substrate interface.

**Figure 10:**
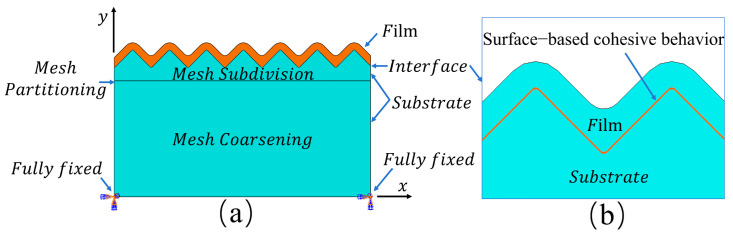
Tungsten thin film–aluminum oxide ceramic finite element model and cohesive zone contact schematic: (**a**) Boundary conditions and mesh requirements for the simulation model; (**b**) cohesive zone model at the interface.

The numerical model was constructed in ABAQUS CAE 2022 software with a global width of 200 µm encompassing over 14 complete microgroove periods and a sufficient substrate depth of 100 µm to eliminate potential bottom boundary effects. The coating thickness was treated as a variable and set to 2 µm, 3 µm, 4 µm, and 5 µm to examine its influence on thermal stress distribution. The geometric relationship of the coating–substrate interface is shown in Figure 10b.

The material properties of the tungsten thin film and the alumina substrate are detailed in Table 4. While the mechanical properties of these refractory materials exhibit inherent temperature dependence, the variations in Young’s modulus and Poisson’s ratio remain relatively limited within the 20 °C to 400 °C thermal regime. Related studies have investigated the thermomechanical stability of these materials and provided a quantitative basis for the modeling parameters. Specifically, empirical data for sintered alumina indicates that the Young’s modulus undergoes a marginal reduction of approximately 4.8% from 416 GPa to 396 GPa over this temperature range, while the Poisson’s ratio shift is limited to a range between 0.231 and 0.235 [24]. For the tungsten film, the recent first principles study by Peng, J. et al. demonstrates that the reduction in Young’s modulus is less than 3% at 400 °C, with the Poisson’s ratio remaining exceptionally stable near 0.298 [25]. Considering that these thermal sensitivities are significantly smaller than the stress variations induced by the CTE mismatch, utilizing these parameters as effective representative values allows the model to accurately characterize the interfacial stress distribution trends while maintaining computational efficiency.

The simulation employed a thermomechanical coupled analysis module to comprehensively account for the nonlinear influence of temperature gradients on the material’s thermal expansion behavior.

The boundary conditions were established to simulate the actual clamping state with the entire bottom surface of the substrate fully fixed. The thermal load simulated uniform heating from 20 °C representing room temperature to the 400 °C deposition temperature and was applied to the entire model. For mesh generation, the CPS4RT 4 node bilinear plane stress thermally coupled element was selected. To enhance computational accuracy, local mesh refinement was applied to the tungsten film and the interface proximity with a minimum element size of 0.2 µm to capture stress gradients. This strategy yielded approximately 210,000 to 225,000 elements for the alumina substrate and 12,000 to 13,000 elements for the tungsten thin film, depending on the microgroove spacing. To rigorously verify grid independence, sensitivity analyses were conducted comparing minimum mesh sizes of 0.1 µm and 0.2 µm. The comparative results demonstrated that the maximum interfacial von Mises stress deviation remained below 2%, which confirms that the 0.2 µm mesh density provides fully converged results.

Regions in the substrate far from the interface exhibit relatively smooth stress changes; therefore, a graded mesh was used for coarse partitioning, with element sizes gradually transitioning from 0.2 µm to 2 µm. This approach effectively enhances computational efficiency while ensuring result convergence. Interfacial contact between the coating and the substrate was modeled using surface-based cohesive behavior to describe interfacial adhesion and damage evolution. A linear elastic constitutive model was adopted for the tungsten coating, while an elastoplastic constitutive model with linear hardening was used to describe the behavior of the alumina substrate.

(3) $$\sigma =\left\{ \begin{array}{cc} E_{s}\varepsilon , & \varepsilon <\frac{\sigma_{y}}{E_{s}}\\ \sigma_{y}+E_{s}^{*}\left(\varepsilon -\frac{\sigma_{y}}{E_{s}}\right), & \varepsilon \geq \frac{\sigma_{y}}{E_{s}} \end{array}\right.$$

where $E_{s}$ is the elastic modulus, $\sigma_{y}$ is the yield strength, $E_{s}^{*}$ is the hardening modulus, ε denotes the strain, and σ represents the corresponding stress.

**Figure 11:**
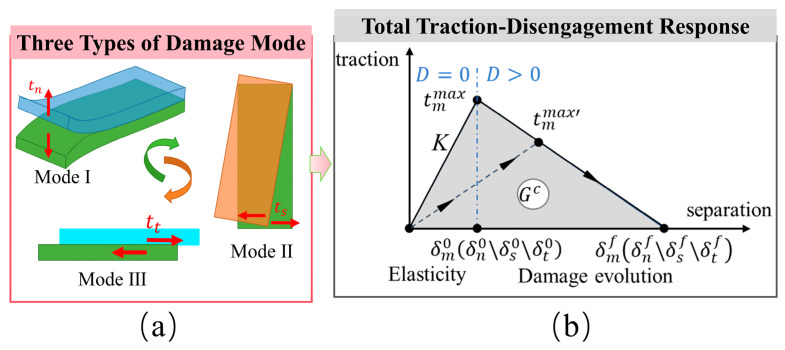
Schematic of (**a**) three damage modes and (**b**) total traction–separation response based on the cohesive zone model.

To characterize the influence of the ceramic substrate’s surface microstructure on the adhesion behavior of the tungsten film, this study introduced a Cohesive Zone Model (CZM) based on the traction–separation law in the finite element model. This model enables simulation of the complete interfacial failure process, from damage initiation to final separation, and has been widely adopted in studies of coating–substrate adhesion [30]. At the coating–substrate interface, a surface-based cohesive interaction was defined, with its constitutive behavior represented by a bilinear traction–separation law accounting for both normal and tangential responses, as shown in Figure 11b. In the linear elastic stage, the stress–displacement relationship can be expressed as [31]

(4) $$t_{m}=\left\{\begin{array}{c} t_{n}\\ t_{s}\\ t_{t} \end{array}\right\}=\left[\begin{array}{ccc} K_{nn} & K_{ns} & K_{nt}\\ K_{ns} & K_{ss} & K_{st}\\ K_{nt} & K_{st} & K_{tt} \end{array}\right]\left\{\begin{array}{c} \delta_{n}\\ \delta_{s}\\ \delta_{t} \end{array}\right\}=K\delta_{m}\;\;\;(\delta_{m}<\delta_{m}^{0})$$

Assuming the interface material is mechanically independent (isotropic) in all three directions, it is generally postulated that there is no coupling between the directions; thus, the relationship is

(5) $$K_{ns}=K_{nt}=K_{st}=0$$

At this point, the stiffness matrix *K* can be simplified to a diagonal form.

(6) $$\left[\begin{array}{ccc} K_{n} & 0 & 0\\ 0 & K_{s} & 0\\ 0 & 0 & K_{t} \end{array}\right]$$

where the normal separation displacement $\delta_{n}>0$. $t_{n}$ and $t_{t}$ represent the normal and tangential separation stresses, respectively, and δ and T denote the displacement and maximum separation stress (i.e., strength), respectively. The subscripts n and s refer to the normal and tangential directions, while the superscripts 0 and f represent the critical displacement corresponding to the strength *T* (damage initiation) and the fracture displacement corresponding to complete failure, respectively.

When the applied load increases beyond the critical value, the interface begins to soften and degrade, meaning the interface enters a damaged state. Typically, damage initiation occurs when a certain criterion is met. For the mixed-mode condition shown in Figure 11b, the quadratic nominal stress criterion is used for judgment, which is described as [32]

(7) $${\left(\frac{\left\langle t_{n}\right\rangle }{t_{n}^{max}}\right)}^{2}+{\left(\frac{t_{s}}{t_{s}^{max}}\right)}^{2}+{\left(\frac{t_{t}}{t_{t}^{max}}\right)}^{2}=1$$

where ⟨ ⟩ represents the Macauley bracket, defined as ⟨t⟩=(|t|+t)/2. It is generally assumed that pure compressive deformation or stress states do not initiate damage. Assuming damage occurs at the interface when the above equation is satisfied, and based on the total displacement of the hybrid mode being

(8) $$\delta_{m}=\sqrt{{\left\langle \delta_{n}\right\rangle }^{2}+\delta_{s}^{2}+\delta_{t}^{2}}$$

A single scalar damage variable $D_{*}$ is introduced to describe interfacial degradation [33]

(9) $$D_{*}=\{\begin{array}{c} \;\;\;\;\;\;\;\;\;\;\;\;\;\;\;0,\;\;\;\;\;\;\;\;\;\;\;\;\;\;\;\;\;\;\;\;\;\;\;\;\;\;\;\;\;\;\;\;\;\;\delta_{m}\leq \delta_{m}^{0}\\ \frac{\delta_{m}^{f}\left(\delta_{m}^{max}-\delta_{m}^{0}\right)}{\delta_{m}^{max}\left(\delta_{m}^{f}-\delta_{m}^{0}\right)},\;\;\;\;\;\;\;\;\;\;\;\;\;\;{\delta_{m}^{0}<\delta }_{m}<\delta_{m}^{f}\\ \;\;\;\;\;\;\;\;\;\;\;\;\;\;\;1,\;\;\;\;\;\;\;\;\;\;\;\;\;\;\;\;\;\;\;\;\;\;\;\;\;\;\;\;\;\;\;\;\;\;\delta_{m}\geq \delta_{m}^{f} \end{array}$$

After introducing the single damage variable $D_{*}\in [0,1]$, cohesive force is described using a linear degradation model. The total interfacial traction force t satisfies the following relationship with the traction force $t_{m}$ in the undamaged state.

(10) $$t=\left(1-D_{*}\right)t_{m}$$

where $D_{*}$ = 0 indicates an intact interface, and $D_{*}$ = 1 indicates complete failure. This linear degradation model assumes that the load-bearing capacity of the interface material decreases linearly with the increase in equivalent displacement within the damage evolution range (${\delta_{m}^{0}<\delta }_{m}<\delta_{m}^{f})$. Since both Mode II and Mode III are classified as shear loading acting on the interface, and their material parameters can often be treated as equivalent, they are commonly unified into a single shear mode in the analysis. To highlight the primary failure mechanism and simplify the model, only Mode I and Mode II are typically considered to characterize the interface response under normal and tangential loading, respectively. As shown in Figure 11a. Therefore, the interfacial traction force after linear degradation can be expressed as

(11) $$\begin{array}{cc} \{\begin{array}{c} t_{n}={\left(1-D_{*}\right)t}_{n}^{max}\\ t_{t}={\left(1-D_{*}\right)t}_{t}^{max} \end{array} & \left(\delta_{m}\geq \delta^{0}\right) \end{array}$$

The critical fracture energy $G^{c}$ in the normal and tangential directions is [34]

(12) $$G_{n}^{c}=\int_{0}^{\delta_{n}^{f}}td\delta_{n}=\frac{1}{2}t_{m}^{max}\delta_{n}^{f}$$

(13) $$G_{t}^{c}=\int_{0}^{\delta_{t}^{f}}td\delta_{t}=\frac{1}{2}t_{m}^{max}\delta_{t}^{f}$$

The interface fracture energy can be termed the mixed-mode fracture energy, and the BK criterion is employed to describe mixed-mode fracture behavior [35].

(14) $$G_{n}^{c}+\left(G_{t}^{c}-G_{n}^{c}\right){\left\{\frac{G_{t}}{\left({G_{n}+G}_{t}\right)}\right\}}^{\eta }=G^{c}$$

Among these, $G_{n}$ and $G_{t}$ represent the normal and tangential energy release rates, respectively. $G_{n}^{c}$ and $G_{t}^{c}$ denote the critical fracture energies for the corresponding directions, while η is the coupling parameter, set to 1.2 in this study. Complete interface failure occurs when the total energy release rate reaches $G^{c}$. Specific model parameters are listed in Table 5.

### 4.2. Mechanism of Microgroove Structures Enhancing Interfacial Adhesion Properties

The introduction of periodic microgroove structures on the substrate surface significantly enhances the adhesion performance of thin films. This strengthening effect primarily originates from mechanically induced changes in interfacial morphology. The microgroove array significantly increases the actual contact area between the film and the substrate, and forms an effective mechanical interlocking structure, thereby improving the interfacial bond strength $\sigma_{b}$ and the critical interfacial fracture energy $G_{c}$. During the film deposition process, due to the mismatch in the coefficient of thermal expansion (CTE) between tungsten and alumina, residual stresses $\sigma_{R}$ gradually accumulate within the film. The interface energy release rate caused by these residual stresses increases linearly with the film thickness *d*, and this relationship can be described by the following expression [40].

(15) $$G\left(d\right)=\frac{\left(1-v^{2}\right)d\sigma_{R}^{2}}{2E}$$

where E is the Young’s modulus of the thin film, and v is its Poisson’s ratio. Interface failure occurs when the energy release rate, G(d), reaches or exceeds the interface’s critical energy release rate, $G_{c}$. The introduction of the microgroove structure significantly increases the value of $G_{c}$, thereby raising the energy threshold required for crack propagation. Consequently, a thicker tungsten film can be deposited without incurring interfacial delamination. This effect is attributed to the groove structure, which effectively retards the initiation and propagation of interfacial cracks by altering the stress distribution, promoting the crack closure effect, and increasing the tortuosity of the crack propagation path.

### 4.3. Effect of Film Thickness on Stress Distribution at the Same Groove Spacing

**Figure 12:**
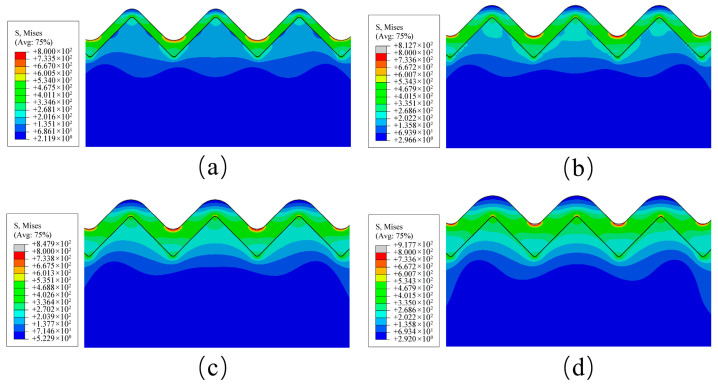
Von Mises stress distribution on films of different thicknesses with substrate groove spacing *D* = 28.8 µm: (**a**) for 2 µm film thickness; (**b**) for 3 µm film thickness; (**c**) for 4 µm film thickness; (**d**) for 5 µm film thickness. The black lines represent the interface between the film and the substrate.

The geometric parameters of the simulation models were strictly defined by the experimental characterization to guarantee physical relevance. The periodic microgroove spacings of 13.6 µm, 24.8 µm, and 28.8 µm established in the numerical analysis were directly extracted from the experimental machining results representing distinct stages of inter-grain interference. By mapping these specific dimensions into the periodic microgroove profile established in Section 4.1.1, the simulation aligns perfectly with the actual dimensional scale of the fabricated interfaces. Building upon this physically consistent model, the influence of film thickness on interfacial stress distribution was investigated by analyzing von Mises stress contours for different film thicknesses on surfaces with a groove spacing of *D* = 28.8 µm, in conjunction with the groove morphology, the dominant influence of film thickness on the interfacial stress distribution was systematically elucidated. Furthermore, comparison with surfaces with *D* = 13.6 µm and *D* = 24.8 µm helped clarify the stress dispersion associated with groove spacing. As shown in Figure 12, the von Mises stress contours for different film thicknesses on the *D* = 28.8 µm surface indicated that, under all thickness conditions, stress concentration consistently occurred at the bottom region of the film grooves, a phenomenon closely associated with the localized stress concentration induced by groove geometry during deposition.

Integrating the fileting process applied to the substrate grooves during model establishment and the physical simulation of the film deposition process, the evolution of stress distribution can be interpreted through the dynamic changes in groove geometry during film growth. When the film is relatively thin, deposition preferentially occurs at the groove bottom, leading to an increased radius of curvature in this region and consequently a moderated stress concentration level. As the film thickness increases, continuous deposition gradually covers the groove sidewalls and ridges. The radius of curvature of the film geometry at the groove bottom progressively decreases, resulting in a pronounced enhancement of stress concentration. From a solid mechanics perspective, this tightened geometric curvature severely restricts the natural volumetric deformation of the deposited material, thereby forcing the thermal strain energy to localize violently at the narrow groove bottoms. This evolution of geometric morphology directly governs the interfacial stress distribution; consequently, the groove bottom becomes the most susceptible region for interfacial failure.

**Figure 13:**
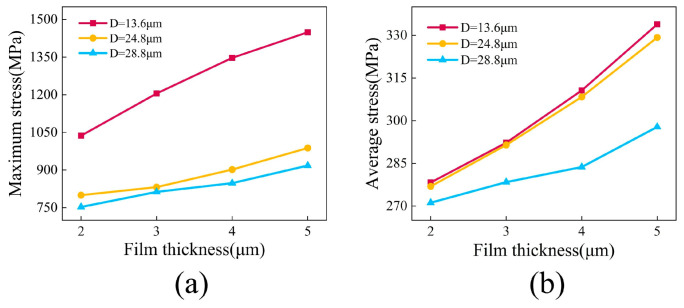
(**a**) Maximum film stress versus film thickness for surfaces with different substrate groove spacings; (**b**) average film stress versus film thickness for surfaces with different substrate groove spacings.

For a quantitative assessment of the stress levels, Figure 13a illustrates the relationship between the maximum film stress and film thickness for the three groove spacing categories. It can be observed that the maximum stress in all groups exhibited a consistent increasing trend with greater film thickness, further confirming the accumulation effect of thermal mismatch stress. Mechanically thicker films possess a greater volume of material that accumulates elastic strain energy under thermal loads, which inherently intensifies the overall driving force for interfacial delamination. Notably, at identical film thicknesses, the maximum stress in the *D* = 28.8 µm group was consistently lower than that in the *D* = 13.6 µm and *D* = 24.8 µm groups. For instance, at a film thickness of 5 µm, the maximum stress in the *D* = 28.8 µm group was approximately 31.8% lower than that in the *D* = 13.6 µm group. This indicates that increasing the groove spacing effectively suppresses local stress peaks and improves the overall stress distribution state. This comparative result fundamentally validates that expanding the spatial topology serves as a highly effective mechanical buffer to dissipate the accumulated thermal strain energy over a wider footprint.

From the perspective of overall stress levels, Figure 13b shows the variation in average stress with film thickness and groove spacing. The average stress increases with film thickness but systematically decreases with larger groove spacing at the same film thickness. For instance, at a film thickness of 4 µm, the average stress for the *D* = 28.8 µm group was 283 MPa, significantly lower than the 310 MPa for the *D* = 13.6 µm group. From a macroscopic perspective this reduction occurs because the expanded trapezoidal geometries increase the effective load-bearing area, which allows the accumulated thermal strain energy to be absorbed and dissipated more evenly. This phenomenon indicates that a larger groove spacing not only helps mitigate local stress concentration but also promotes a more uniform distribution of stress across a broader area, thereby delaying the initiation and propagation of interfacial damage.

### 4.4. Stress Distribution for Identical Film Thickness at Different Groove Spacings

To elucidate the influence of groove spacing on the interfacial stress state, this study fixed the tungsten film thickness at 4 µm and conducted a comparative analysis of the von Mises stress distribution, average stress versus temperature curves, and maximum stress versus spacing curves for the three groove spacings. This approach systematically reveals the regulatory mechanisms of groove spacing on the degree of stress concentration, the thermal mismatch response, and stress uniformity.

**Figure 14:**
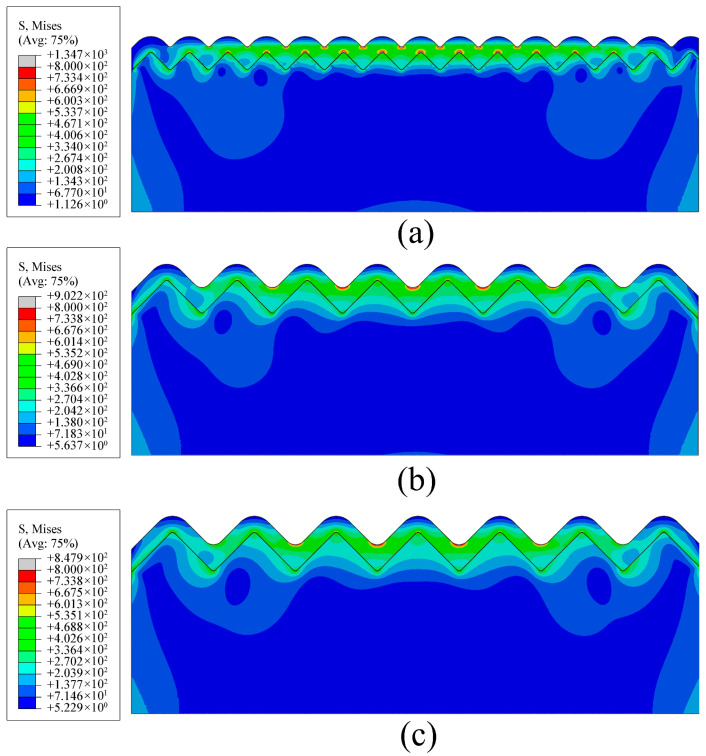
Comparison of von Mises stress distribution on 4 µm thick tungsten films with different substrate groove spacings: (**a**) *D* = 13.6 µm; (**b**) *D* = 24.8 µm; (**c**) *D* = 28.8 µm. The black lines represent the interface between the film and the substrate.

Analysis of the stress contour maps in Figure 14 reveals that as the groove spacing increases from *D* = 13.6 µm to *D* = 28.8 µm, the stress concentration at the groove bottoms exhibits a pronounced alleviation trend. For the *D* = 13.6 µm surface (Figure 14a), distinct red high-stress bands are observed at the groove bottoms, characterized by steep stress gradients and a relatively large region of concentrated stress. At the *D* = 24.8 µm surface (Figure 14b), these high-stress bands become narrower, and the stress gradients are more gradual. For the *D* = 28.8 µm surface (Figure 14c), the high-stress regions are further dispersed, and the stress distribution becomes notably more uniform. The critical importance of achieving such stress uniformity has been emphasized in recent advanced manufacturing research. For instance, cutting edge studies on pre-stress assisted machining mathematically formalize the stress compensation mechanism in their Equation (3), conclusively demonstrating that actively regulating the local stress field is essential for maintaining high surface integrity [41]. Aligning with these fundamental mechanics, our periodic geometric optimization effectively functions as a static stress regulator. From a mechanistic viewpoint, the narrow valleys at the smaller spacing of 13.6 µm act as severe geometric constraints that restrict the natural thermal expansion of the deposited film, forcing the thermal mismatch energy to localize heavily at the bottom. By contrast, the wider trapezoidal structures at the larger spacing of 28.8 µm provide sufficient spatial tolerance, allowing the interfacial materials to accommodate thermal deformation elastically and dissipate the concentrated energy.

**Figure 15:**
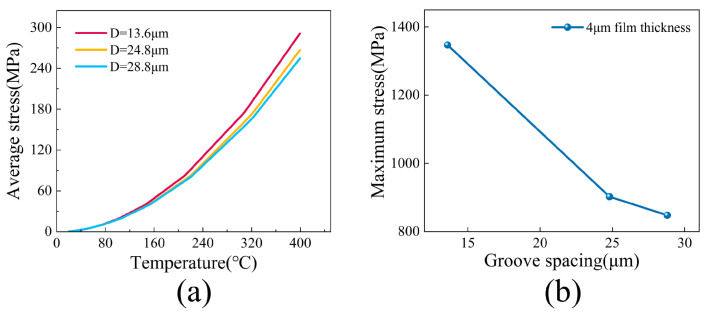
(**a**) Temperature-dependent curve of average interfacial stress at different groove spacings; (**b**) variation curve of maximum interfacial stress with groove spacing for a 4 µm thick tungsten film.

Analysis of the average stress–temperature curves in Figure 15a reveals the distinct influence of groove spacing on the evolution of thermal mismatch stress. During the temperature rise from 20 °C to 160 °C, the average stress growth trends for the surfaces with three distinct groove spacings remain essentially consistent. This behavior is attributed to the relatively small thermal expansion mismatch at lower temperatures, where the stress can be largely accommodated through elastic deformation coordination between the film and substrate. However, once the temperature exceeds 160 °C, the three curves begin to diverge noticeably. The average stress increases most rapidly for the *D* = 13.6 µm surface, followed by the *D* = 24.8 µm surface, while the *D* = 28.8 µm surface exhibits the slowest rate of increase.

This differentiation arises from the regulation of the thermal stress buffering capacity by the groove spacing. The groove–ridge structure with the larger spacing (*D* = 28.8 µm) can absorb part of the thermal mismatch stress through morphological elastic deformation, thereby reducing the growth rate of the average stress. In this expanded configuration, the widened periodic structures function as a compliant mechanical layer that safely flexes to accommodate the expanding film. In contrast, the structure with the smaller spacing (*D* = 13.6 µm) lacks sufficient buffering capacity, causing the thermal mismatch stress to be almost entirely borne by the film, which leads to a steep increase in average stress. From a thermomechanical coupling perspective, this observed pattern highlights the advantage of larger groove spacings in mitigating thermal stress.

The maximum stress versus groove spacing curve in Figure 15b further quantifies the regulatory role of groove spacing from the perspective of peak stress. At a fixed film thickness of 4 µm, the maximum stress shows a monotonic decreasing trend with increasing groove spacing. Specifically, the maximum stress reaches approximately 1350 MPa on the surface with a groove spacing of *D* = 13.6 µm, 900 MPa on the surface with a groove spacing of *D* = 24.8 µm, and 850 MPa on the surface with a groove spacing of *D* = 28.8 µm. This result clearly demonstrates the inverse relationship between groove spacing and the stress concentration factor. Enlarging the groove spacing effectively reduces the stress concentration factor by expanding stress dispersion pathways and decreasing the local morphological curvature, thereby leading to a substantial reduction in maximum stress. Furthermore, the prominent nonlinear decline in peak stress perfectly corroborates the geometric saturation trend discussed earlier, indicating that once the ridge isolation is achieved, the interfacial mechanical optimization approaches a highly stable theoretical limit.

### 4.5. Stress Distribution at the Interface for the Same Film Thickness with Different Groove Spacing

To further elucidate the regulatory mechanism of microgroove spacing on the interfacial stress state, a systematic analysis was performed on the results of the thermomechanical coupled simulations conducted for the 4 µm tungsten film on surfaces with three different groove spacings, with a focused examination of the stress distribution characteristics within the interfacial region.

**Figure 16:**
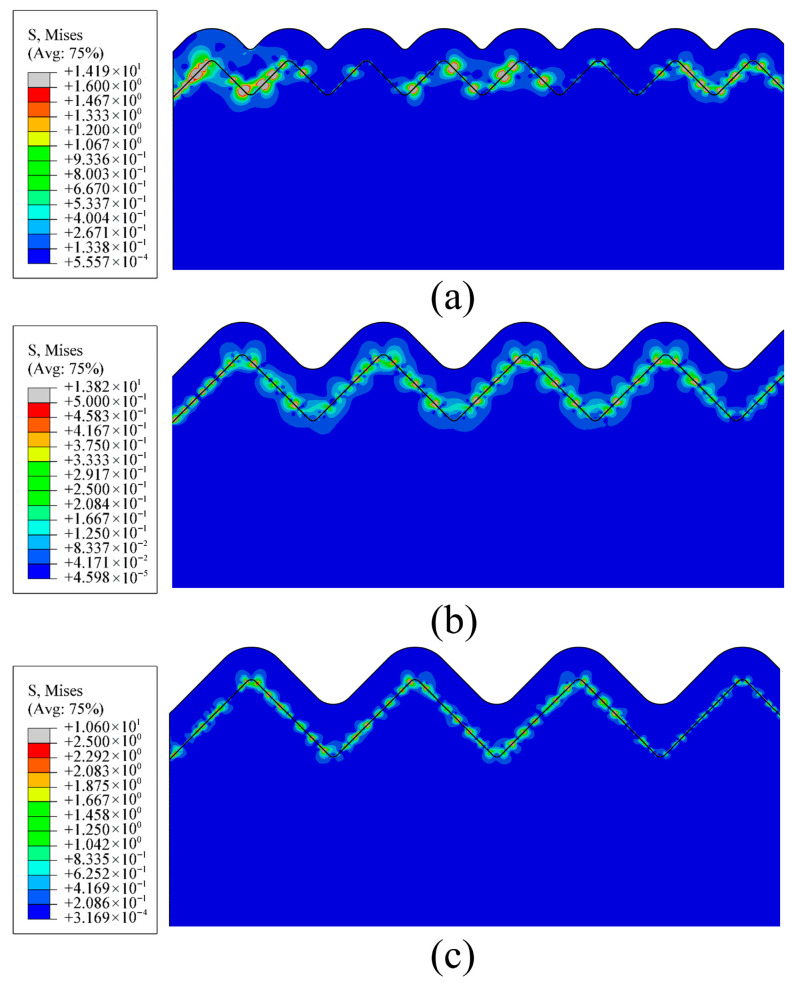
Comparison of von Mises stress contour maps at the interface for different substrate groove spacings under a 4 µm thick film: (**a**) *D* = 13.6 µm; (**b**) *D* = 24.8 µm; (**c**) *D* = 28.8 µm. The black lines represent the interface between the film and the substrate.

The stress contour maps in Figure 16 indicate that both the degree of stress concentration and the density of its distribution at the interface exhibit a significant decreasing trend as the groove spacing increases. In the stress contour corresponding to the *D* = 13.6 µm surface (Figure 16a), distinct high-stress concentration zones appear at the interface, demonstrating a pronounced localization of stress distribution. Under the *D* = 24.8 µm surface (Figure 16b), the density and extent of these high-stress regions are reduced, leading to an improved uniformity in stress distribution. For the *D* = 28.8 µm surface (Figure 16c), the high-stress areas at the interface become further dispersed, and the stress concentration phenomenon is markedly alleviated.

This phenomenon arises from the regulatory effect of groove spacing on the interface mechanical interlocking and stress transfer pathway relationship. From a mechanistic perspective, the localized stress accumulation at the narrow spacing acts as a mechanical wedge that promotes film delamination, while the broadened periodic configuration at the larger spacing functions as continuous mechanical anchors that efficiently neutralize thermal mismatch effects.

**Figure 17:**
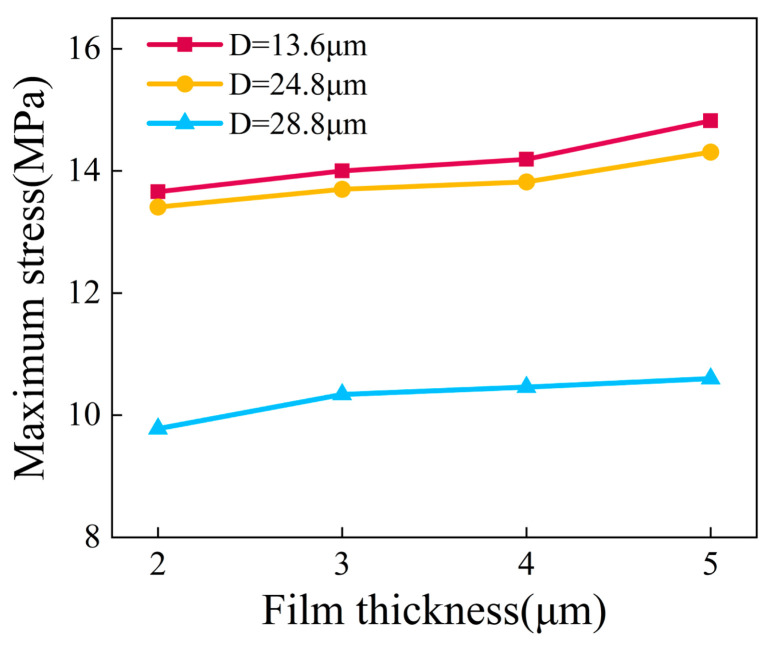
Curve of maximum interfacial stress versus tungsten film thickness at different groove spacings.

By extracting the maximum interfacial stress at different film thicknesses (Figure 17), the synergistic effect of groove spacing and film thickness on interfacial stress can be further clarified. For the surface with a groove spacing of *D* = 13.6 µm, the maximum interfacial stress increases from approximately 13.5 MPa to 14.8 MPa as the film thickness grows from 2 µm to 5 µm. A similar trend is observed for the *D* = 24.8 µm surface, where the maximum stress rises from about 13.2 MPa to 14.3 MPa. In contrast, the *D* = 28.8 µm surface exhibits substantially lower stress levels. Although the maximum interfacial stress also increases with film thickness, the growth rate is more moderate, rising from approximately 9.8 MPa to 10.6 MPa.

This consistent stress evolution can be attributed to the dual mechanisms of thermal mismatch stress accumulation and geometric stress mitigation. Increased film thickness causes a linear growth in the energy release rate G(d), as described in Equation (15), which drives the overall rise in interfacial stress and explains the experimental observation that thicker films are more prone to delamination. However, the finite element simulation provides a direct mechanistic explanation for the adhesion enhancement observed in experiments by verifying that expanding the groove spacing effectively counteracts this accumulation effect. The numerical results demonstrate that the structural optimization at *D* = 28.8 µm yields a substantial reduction in the maximum interfacial stress by broadening the stress diffusion pathways. This quantitative stress relaxation directly validates the higher critical interfacial fracture energy measured in the experimental characterization and confirms that increasing the microgroove spacing is a highly effective strategy to secure the structural integrity of the deposited thin films.

From the perspective of the microscopic mechanism of interfacial failure, high stress concentration at the interface serves as the primary trigger for crack initiation and propagation. The high-stress density zones at the *D* = 13.6 µm and *D* = 24.8 µm surfaces readily act as “sensitive sites” for crack initiation, with increasing film thickness further amplifying this risk. In contrast, the relatively low stress levels and uniform distribution at the *D* = 28.8 µm surface significantly enhance the interfacial damage tolerance, maintaining a low probability of crack initiation even under thick-film conditions. Ultimately this confirms that optimizing the surface topology successfully transforms a highly vulnerable interface into a robust structural foundation capable of sustaining severe thermal fluctuations.

## 5. Conclusions

This research successfully achieved the general objective of enhancing the interfacial adhesion between tungsten thin films and alumina ceramic substrates through the implementation of a deterministic surface microgroove design. The specific experimental objective was fulfilled by establishing a quantitative correspondence between surface roughness and periodic micro geometry based on the CNC scratching offset distances. By utilizing the relationship between the target roughness *Ra* and the groove depth *h*, the stochastic nature of the substrate surface was transformed into reproducible periodic structures. This deterministic approach enables the surface morphology to be accurately captured in numerical analysis, which bridges the gap between random surface topographies and idealized geometric models. The experimental results confirm that regulating the transverse spacing of the scratching paths effectively governs the transition of the surface morphology and ensures the successful fabrication of microtextures with stable geometric characteristics.

The numerical objective was accomplished through thermomechanical simulations, which quantified the stress mitigation mechanism for the three distinct surface categories. The analysis reveals that expanding the microgroove spacing to 28.8 µm reduces the maximum interfacial stress by 31.8% and the average film stress by 10% compared to the 13.6 µm spacing. This integrated finding clarifies how the structural optimization redistributes thermal mismatch stresses and delays the initiation of film delamination, which directly validates the superior interfacial adhesion measured in the experiments. Consequently, the achievement of these objectives provides a rigorous methodology for the structural design of high-performance ceramic metal systems in extreme environments.

To build upon these findings, future research will be approached in a clearly defined, staged manner. The first stage will focus on investigating the durability behavior of these microstructures under complex thermomechanical cycling to ensure long-term stability. The second stage will involve the design and characterization of hierarchical structures to further enhance interfacial reliability at multiple length scales.

## Figures and Tables

**Table 1:** Relationship between single-grain scratch depth and processing parameters.

Experiment Number	Axial Load (N)	Feed Rate (mm/min)	Scratch Length (mm)	Scratch Depth (µm)
1	20	200	10	5
2	40	200	10	12
3	60	200	10	20
4	80	200	10	23
5	100	200	10	25

**Table 2:** Relationship between scratch depth and surface roughness of multi-abrasive structures and offset distance.

Experiment Number	Groove Spacing (µm)	Axial Load (N)	Scratch Depth (µm)	Surface Roughness *Ra* (µm)
1	13.6	60	10	1.7
2	24.8	60	18	3.1
3	28.8	60	20	3.7

**Table 3:** Correspondence table of surface roughness and microgroove geometric parameters for alumina ceramic substrates.

*Ra* (µm)	*h* (µm)	*D* (µm)
1.7	6.8	*D* = 13.6
3.1	12.4	*D* = 24.8
3.6	14.4	*D* = 28.8

**Table 4:** Material performance parameters table [26,27,28,29].

Materials	Young’s Modulus (GPa)	Poisson Ratio	CTE (10^−6^/°C)	Thermal Conductivity (W/m·K)
Tungsten film	410	0.28	4.6	173
Alumina substrate	370	0.22	8	30

**Table 5:** Finite element analysis parameters [10,36,37,38,39].

Parameter Name	Value
Initial composite stiffness (K)	4.1 × 10^6^ N/mm^3^
$\mathrm{Initial}\;\mathrm{Interface}\;\mathrm{Normal}\;\mathrm{Strength}\;\left(t_{n}^{max}\right)$	50 MPa
$\mathrm{Initial}\;\mathrm{Interfacial}\;\mathrm{Tan}\mathrm{gential}\;\mathrm{Strength}\;\left(t_{t}^{max}\right)$	80 MPa
$\mathrm{Critical}\;\mathrm{Normal}\;\mathrm{Fracture}\;\mathrm{Energy}\;\left(G_{n}^{c}\right)$	380 J/m^2^
$\mathrm{Critical}\;\mathrm{Tan}\mathrm{gential}\;\mathrm{Fracture}\;\mathrm{Energy}\;\left(G_{t}^{c}\right)$	210 J/m^2^

## Data Availability

The authors declare that all data supporting the findings of this study are available within the article.
